# Potential anti-cancer effect of N-hydroxy-7-(2-naphthylthio) heptanomide (HNHA), a novel histone deacetylase inhibitor, for the treatment of thyroid cancer

**DOI:** 10.1186/s12885-015-1982-6

**Published:** 2015-12-23

**Authors:** Seok-Mo Kim, Ki-Cheong Park, Jeong-Yong Jeon, Bup-Woo Kim, Hyeung-Kyoo Kim, Ho-Jin Chang, Seung-Hoon Choi, Cheong-Soo Park, Hang-Seok Chang

**Affiliations:** Department of Surgery, Thyroid Cancer Center, Gangnam Severance Hospital, Yonsei University College of Medicine, 211 Eonjuro, Gangnam-gu, Seoul 135-720 South Korea; Department of Nuclear Medicine, Yonsei College of Medicine, Seoul, 120-752 South Korea

## Abstract

**Background:**

Thyroid cancer has been indicated to have a higher global proportion of DNA methylation and a decreased level of histone acetylation. Previous studies showed that histone gene reviser and epigenetic changes role significant parts in papillary and anaplastic thyroid cancer tumorigenesis. The goal of this research was to study the endoplasmic reticulum (ER) stress-mediated actions of the dominant histone deacetylase (HDAC) inhibitor, N-hydroxy-7-(2-naphthylthio) hepatonomide (HNHA), in thyroid cancer and to explore its effects on apoptotic cell death pathways.

**Methods:**

Experiments were achieved to conclude the effects of HNHA in papillary thyroid cancer (PTC) and anaplastic thyroid cancer (ATC) cell lines and xenografts, as compared with two other established HDAC inhibitors (SAHA; suberoylanilide hydroxamic acid and TSA; trichostatin A).

**Results:**

Apoptosis, which was induced by all HDAC inhibitors, was particularly significant in HNHA-treated cells, where noticeable B-cell lymphoma-2 (Bcl-2) suppression and caspase activation were observed both in vitro and in vivo. HNHA increased Ca^2+^ release from the ER to the cytoplasm. ER stress-dependent apoptosis was induced by HNHA, suggesting that it induced caspase-dependent apoptotic cell death in PTC and ATC. PTC and ATC xenograft studies demonstrated that the antitumor and pro-apoptotic effects of HNHA were greater than those of the established HDAC inhibitors. These HNHA activities reflected its induction of caspase-dependent and ER stress-dependent apoptosis on thyroid cancer cells.

**Conclusions:**

The present study indicated that HNHA possibly provide a new clinical approach to thyroid cancers, including ATC.

## Background

Thyroid cancer is the most commonly occurring endocrine malignancy and its incidence has increased steadily over the past three decades worldwide [1, 2]. Generally, thyroid cancer can be treated effectively with surgery or radioactive iodine [3]. ATC is the least common, but the most aggressive, of all thyroid cancers [4]. The mechanisms driving the progress of ATC are not completely understood. ATCs are currently treated with chemotherapy, radiotherapy, and/or surgery [4, 5]. Nevertheless, patients with ATC only have a median survival of 5 months and less than 20 % survive for 1 year after diagnosis [6]. Early tumor dissemination occurs in this type of cancer, resulting in 40 % of patients showing distant metastases and 90 % showing invasion of adjoining tissue on presentation [7]. The present study investigated HDAC inhibitors as a novel chemotherapy for PTC and ATC. HDACs are often highly expressed in cancer cells [8–10]. These enzymes restrain the transcription of tumor suppressor genes and so offer bright targets for cancer therapy [11, 12]. HDAC inhibitors are a group of small molecules that accelerate gene transcription by reducing HDAC activity, inducing chromatin remodeling; these inhibitors have been extensively studied as potential drugs for treating cancer [12–15]. HDAC inhibitors affect various well-known features of cancer cells, involving apoptosis, autophagy, growth inhibition and differentiation [16–18]. They are extremely specific for cancer cells over normal cells, owing to their induction of pro-apoptotic genes and ER stress, in addition to their effects on DNA repair mechanisms [19, 20]. HNHA is a dominant HDAC inhibitor that was previously shown to drive histone acetylation and downregulate the expression of HDAC target genes [21, 22]. HNHA showed powerful anti-cancer activity in breast cancer cells and fibrosarcoma [21–23]. Here, we researched this dominant HDAC inhibitor and its ER stress-mediated roles in thyroid cancer and explored the effects of HNHA on apoptotic cell death pathways in PTC and ATC.

## Methods

### Cell culture

The patient-derived thyroid cancer cell lines, SNU-80 (ATC) and SNU-790 (PTC), were purchased from the Korea Cell Line Bank (Seoul National University, Seoul, Korea) and cultured in RPMI-1640 medium with 10 % fetal bovine serum. The cells lines were authenticated by short tandem repeat profiling, karyotyping and isoenzyme analysis. Ethics approval about patient-derived thyroid cancer cell lines was approved by the Institutional Review Board (IRB) of Seoul National University hospital (Seoul, Republic of Korea).

### Cell viability assay

Cell viability was measured by 3-(4,5-Dimethylthiazol-2-yl)-2,5-Diphenyltetrazolium Bromide (MTT) assay. Cells were cultured and grown to accomplish 70 % confluency. The indicated drugs were added to achieve final concentrations of 0-100 μM. Cells were then incubated for the indicated times prior to determination of cell viability by MTT assay. Data were indicated as a proportion of the signal surveyed in vehicle-treated cells and shown as the mean ± standard error of the mean (SEM) of triplicate experiments.

### Evaluation of apoptotic cell death

Analysis of apoptosis and then identified with a TUNEL (terminal deoxynucleotidyl transferase dUTP nick end labeling) kit (Promega, Madison, WI, USA). Images of the total and apoptotic cells (fluorescent green) were assembled with a confocal microscope (LSM Meta 700; Carl Zeiss, Oberkochen, Germany) and analyzed with the Zeiss LSM Image Browser software, version 4.2.0121.

### Cytosolic free Ca^2+^ measurements by microspectrofluorimetry

The intracellular Ca^2+^ levels in SNU-80 and SNU-790 cells were imaged using a Ca^2+^-sensitive fluorescent dye, Fura-2 AM. Fluorescence intensities (∆F) were normalized to those recorded in resting cells.

### Immunoblot analysis

The antibodies for histone H3 and acetyl-histone H3, α-tubulin and acetyl-α-tubulin, p53 and p21 were obtained from Abcam (Cambridge, UK). Apaf-1, CDK 4, CDK 6, cyclin D1, Bcl-2, p-NFκB, caspase-3, caspase-9 and β-actin antibodies were purchased from Santa Cruz Biotechnology (Santa Cruz, CA, USA). Antibodies for GRP78, ATF4, CHOP, PERK, p-PERK, eIF2α and p-eIF2α were purchased from Cell Signaling Technology (Danvers, MA, USA). The Bax antibody was obtained from Novus Biologicals (Littleton, CO, USA).

### Flow cytometry analysis of the cell cycle

Cell cycle dispersion was then analyzed with a FACS Calibur Flow Cytometer (BD Biosciences, San Jose, CA, USA). The proportions of cells in the G_0_/G_1_, S and G_2_/M phases were analyzed by FlowJo v8 software for MacOSX (Tree Star, Ashland, OR, USA).

### Electrophoretic mobility shift assay (EMSA)

The DNA binding effect of NFκB to the Bcl-2 promoter was investigated using a ^32^P-labeled oligonucleotide encoding the NFκB transcription factor binding sites found in the Bcl-2 promoter region. Oligonucleotides including the consensus-binding site for NFκB (GATCGAGGGGACTTTCCCTACG) were 5′-end labeled with ɣ-^32^P-dATP and polynucleotide kinase. Nuclear proteins (5 μg) were incubated with 1 μl of labeled oligonucleotide (20,000 c.p.m.) in 20 μl incubation buffer for 20 min at room temperature.

### Human thyroid cancer cell xenograft

Human thyroid cancer cells (2.0 × 10^7^ cells/mouse) were injected subcutaneously into female BALB/c nude mice. After 7 ~ 10 days, mice were grouped randomly (*n* = 10/group) and injected intraperitoneally with 25 mg/kg SAHA, TSA or HNHA once every 2 days for a total of ten injections. Tumor size was measured by calipers. Tumor volume was calculated using the following formula: L × S^2^/2 (where L was the longest diameter and S was the shortest diameter). All in vivo experiments were conducted with the permission of the Animal Experiment Committee of Yonsei University.

### In vivo toxicity study

In vivo toxicity was investigated in female BALB/c nude mice. Every group of 10 mice was treated intraperitoneally with HNHA, SAHA or TSA at a dose of 25 mg/kg. Five animals were housed in each cage and they were observed regularly for external signs of lethality or toxicity. The conditions were controlled to provide 12 h light and 12 h darkness, at a temperature of 22 °C, with 40–60 % humidity. Membrane-filter purified and autoclaved tap water and standard diet of rodent pellets were provided ad libitum.

### Immunohistochemistry

Immunohistochemical staining was performed using a standard protocol. Primary monoclonal antibodies directed Ki-67 (Abcam, Cambridge, UK) were diluted in PBS at a ratio of 1:100. Mayer’s hematoxylin as a counterstain in all tissue sections.

### Statistical analysis

Statistical analysis was performed with GraphPad Prism software (GraphPad Software Inc., La Jolla, CA, USA). One-way ANOVA was performed for the multi-group analysis, and two-tailed Student’s t-tests were used for two-group analyses. Values are indicated as means ± SEM. *P* values < 0.05 were regarded as statistically significant.

## Results

### HNHA inhibited the proliferation of ATC and PTC cells

**Fig. 1:**
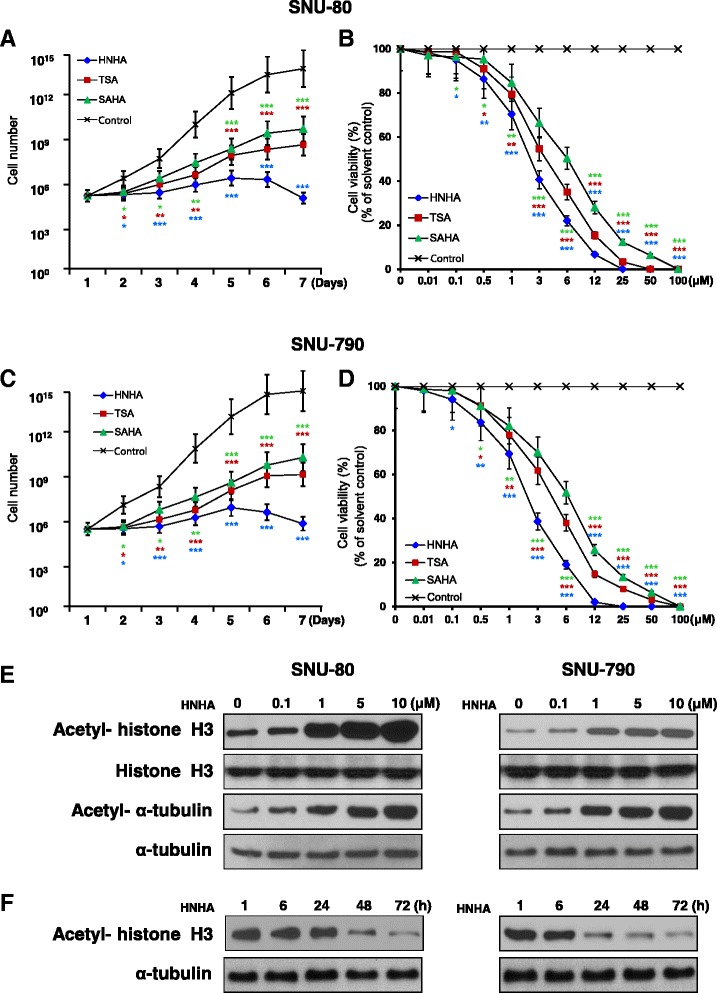
Histone deacetylase inhibitors suppressed proliferation of anaplastic thyroid cancer (ATC) and papillary thyroid carcinoma (PTC) cell lines. Cell viability and proliferation assays demonstrated that HNHA caused the greatest inhibition of thyroid cancer cell proliferation in SNU-80 ATC (**a** and **b**) and SNU-790 PTC cells (**c** and **d**). TSA, trichostatin A; SAHA, suberoylanilide hydroxamic acid. Data points indicate the mean % of the value observed in the solvent-treated control. All tests were repeated three times and the data symbolize the mean ± standard deviation. SNU-80 and SNU-790 cells were treated for 24 h with the expressed concentrations of HNHA (**e**) or with 15 μM HNHA for the indicated time-periods (**f**) prior to isolation of total protein and evaluation of histone H3 and α-tubulin acetylation by immunoblotting. *P < 0.05 vs. Control, **P < 0.01 vs. Control, ***P < 0.005 vs. Control

To investigate the anti-cancer activity of HNHA alongside two well-known HDAC inhibitors (TSA and SAHA), we assayed ATC (SNU-80) and PTC (SNU-790) cell proliferation in the presence and absence of these compounds using an MTT assay (Table 1). HNHA had a lower half-maximal inhibitory concentration (IC_50_) than TSA and SAHA in ATC and PTC cells. Further characterization of the effects of HDAC inhibitors on ATC and PTC cell viability showed that they all reduced the viability of ATC and PTC cells, as compared to vehicle control-treated cells. However, HNHA provided the most significant suppression of cell proliferation (Fig. 1a and c) and this effect was concentration-dependent (Fig. 1b and d).

**Table 1:** Half-maximal inhibitory concentration (IC_50_) values were determined using a cell proliferation assay

Cell line	Histopathology	Animal	Cell proliferation (IC_50_*) (μM)		
			HNHA	TSA	SAHA
SNU-80	Thyroid: anaplastic	Human	2.74 (±0.9)^*^	4.02 (±1.0)	6.74 (±1.1)
SNU-790	Thyroid: papillary	Human	2.32 (±1.0)^*^	3.91 (±1.2)	5.31 (±1.4)

Each data point denotes the mean of three independent IC_50_ values calculated from triplicate MTT assays

*SD* standard deviation

*reflect that were most significantly different between the groups

### HNHA induced histone H3 acetylation in ATC and PTC

We exposed ATC (SNU-80) and PTC (SNU-790) cells to various concentrations of HNHA and then estimated histone H3 acetylation by immunoblotting. Acetylation of α-tubulin and histone H3 were induced by HNHA in a concentration-dependent manner (Fig. 1e). Histone H3 acetylation climaxed at 1 h after exposure to HNHA and remained stable for 48 h (Fig. 1f). These result indicated that HNHA could induce non-histone proteins, as well as stable acetylation of histone H3, in ATC and PTC. Furthermore, HNHA produced concentration-dependent cytotoxicity and induced greater reductions in cell viability at low concentrations (2.32 ± 1.0 μM in SNU-790; 2.74 ± 0.9 μM in SNU-80) than did SAHA (5.31 ± 1.4 μM in SNU-790; 6.74 ± 1.1 μM in SNU-80) or TSA (3.91 ± 1.2 μM in SNU-790; 4.02 ± 1.0 μM in SNU-80).

### ER stress-induced release of cytoplasmic free Ca^2+^ was increased by HNHA

**Fig. 2:**
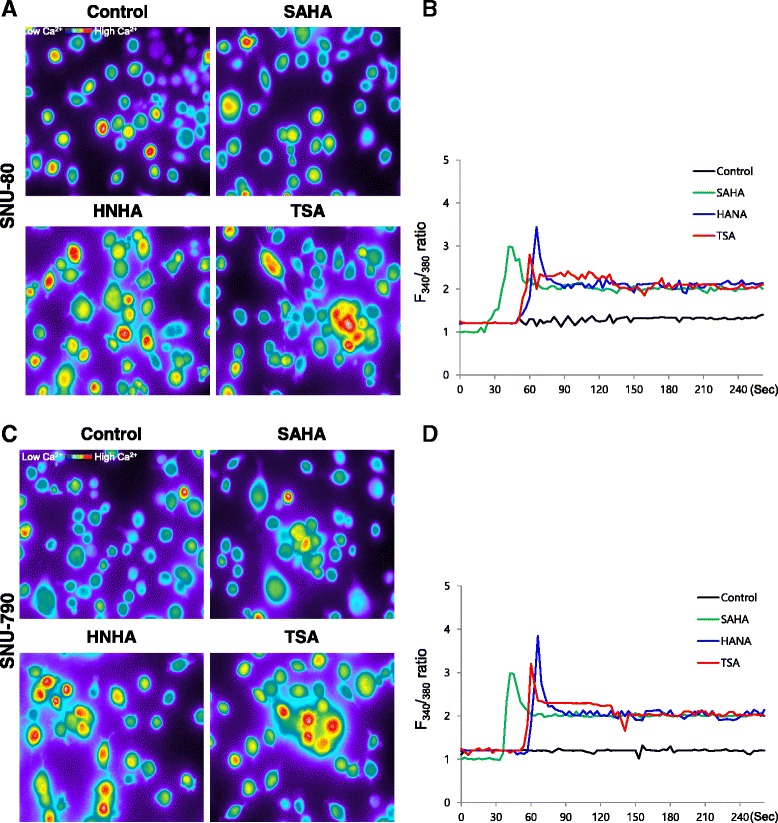
Cytosolic free Ca^2+^ measurements by microspectrofluorimetry in ATC and PTC cells exposed to histone deacetylase inhibitors. Ca^2+^ response in Fura 2 AM-loaded ATC (**a** and **b**) and PTC (**c** and **d**) cells after treatment with SAHA, TSA or HNHA

We measured the change in intracellular Ca^2+^ levels using microspectrofluorimetry. As shown in Fig. 2, the intracellular Ca^2+^ level increased in HDAC inhibitor-treated cells, as compared with control cells (Fig. 2a and c). The cytoplasmic Ca^2+^ levels in HDAC inhibitor-treated cells failed to return to the basal levels observed in control cells (Fig. 2b and d).

### HNHA induced ER stress-dependent cell cycle arrest in ATC and PTC

**Fig. 3:**
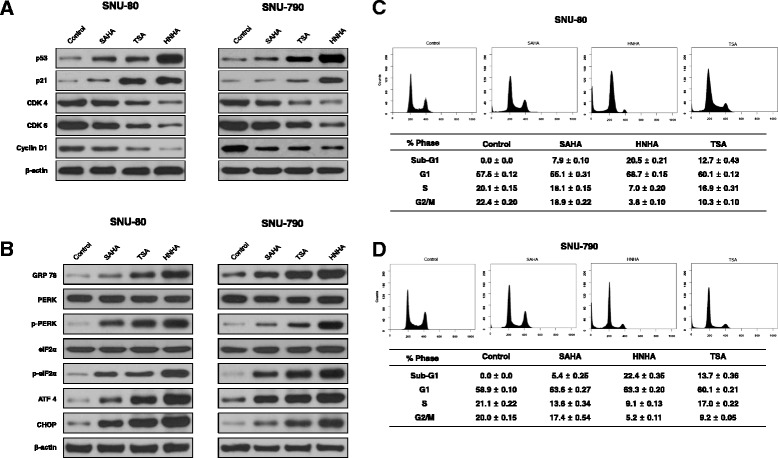
Histone deacetylase inhibitors induced cell cycle arrest and endoplasmic reticulum stress in ATC and PTC cells. Immunoblot analysis of the indicated cell lines following exposure to SAHA, TSA or HNHA showed that HNHA potently induced the expression of cell cycle arrest proteins and reduced expression of positive regulators of the cell cycle (**a**). SNU-80 and SNU-790 were exposed to the indicated inhibitors for 24 h prior to analyzing the expression of GRP 78, ATF 4, CHOP, PERK, p-PERK, eIF2α and p-eIF2α (markers of endoplasmic reticulum stress) by immunoblotting (**b**). Cells were exposed to the indicated inhibitors, harvested and stained with propidium iodide prior to analysis by flow cytometry and FlowJo v8 software (**c** and **d**)

Immunoblot analyses of protein levels in ATC (SNU-80) and PTC (SNU-790) cell lines indicated that HNHA induced more marked increases in the levels of p53 and p21, well-known arrestors of the cell cycle, and decreases in the levels of cyclin D1, CDK 4 and CDK 6, positive regulators of the cell cycle, as compared with SAHA or TSA (Fig. 3a). We also tested whether these compounds induced ER stress by treating SNU-80 and SNU-790 with SAHA, TSA or HNHA for 24 h and analyzing the expression of GRP 78, ATF 4, CHOP, PERK, p-PERK, eIF2α and p-eIF2α by immunoblotting (Fig. 3b). The HNHA-treated cells showed the strongest increase in these markers of ER stress. Flow cytometry was used to study the influence of these compounds on cell cycle progression. The HDAC inhibitors increased G_0_/G_1_ phase arrest and enriched the sub-G_0_ population (*p* < 0.05), indicating cell cycle arrest and apoptosis in these ATC and PTC cell lines (Fig. 3c and d). These data suggested that, of the compounds tested, HNHA was the most potent inducer of ER stress. This resulted in ER stress-dependent apoptosis, cell cycle arrest and the strongest inhibition of ATC and PTC cell line viability.

### HNHA induced caspase-dependent apoptosis of ATC and PTC cell lines

**Fig. 4:**
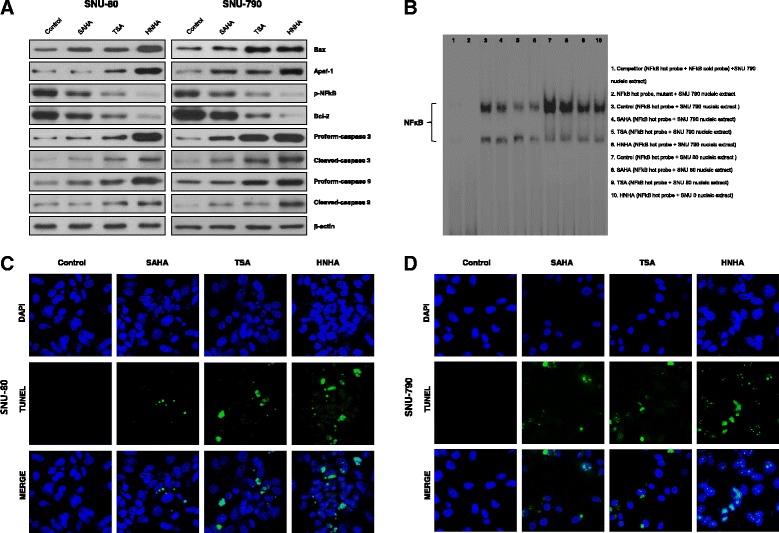
Histone deacetylase inhibitors caussed apoptotic cell death in ATC and PTC cells. Immunoblot analyses suggested that the indicated inhibitors increased the levels of apoptotic proteins and reduced those of anti-apoptotic proteins in ATC and PTC cells (**a**). An electrophoretic mobility shift assay was carried out using a ^32^P-labeled oligonucleotide probe for the NF-κB binding sites on the Bcl-2 promoter (**b**). TUNEL assay of ATC and PTC cells; TUNEL-positive (apoptotic) cells are indicated (× 400) (**c**, **d**)

To research the pro-apoptotic signaling pathways stimulated by exposure of PTC and ATC to HDAC inhibitors, the expression of pro-apoptotic (Bax and Apaf-1) and anti-apoptotic (phosphorylated NF-κB p65 and Bcl-2) members of the Bcl-2 family and the stimulation of caspase-3 and caspase 9, major executioners of apoptosis, were investigated by immunoblotting (Fig. 4a). These results implied that HNHA enhanced the pro-form of caspase-3 and increased the cleavage of pro-caspase-3 and -9 more powerfully than did TSA or SAHA (Fig. 4a).

NF-κB is a transcriptional factor and we investigated the potential p-NF-κB binding sites in the Bcl-2 promoter region. An EMSA (Fig. 4b) identified two bands corresponding to the labeled NF-κB probe following incubation with nuclear extracts of SNU-80 (Fig. 4b, lanes 7-10) or SNU-790 (Fig. 4b, lanes 3-6) cells. The specificity of the EMSA result was proved by complete inhibition of NF-κB probe-DNA binding by excess unlabeled NF-κB probe (Fig. 4b, lane 1). In addition, a like amount of mutated NF-κB probe also foundered to bind (Fig. 4b, lane 2). HNHA-treated cells showed the strongest decrease in NF-κB binding. Together, these results demonstrated that HNHA inhibited Bcl-2 transcription. The TUNEL assay proved that HNHA induced apoptosis in ATC and PTC cell lines more powerfully than did TSA or SAHA (Fig. 4c and d). These data indicated that HNHA is a strong inducer of apoptosis in these ATC and PTC cell lines and that it exerts this effect through inhibition of the Bcl-2 pathway and caspase activation.

### HNHA reduced xenograft growth and improved survival in vivo

**Fig. 5:**
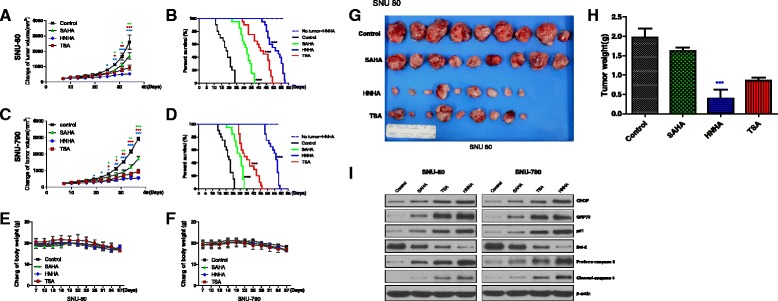
Histone deacetylase inhibitors produced anti-tumor effects in thyroid cancer cell xenografts in vivo. Athymic nude mice with established tumors were treated with the indicated inhibitors. Data represent the mean tumor volumes. HNHA caused more powerful inhibition of tumor developement than did SAHA or TSA and followed in the greatest prolongation of survival in mice with anaplastic thyroid cancer (ATC; **a**, **b**) and papillary thyroid carcinoma (PTC; **c**, **d**) xenografts (*n* = 10 mice/group). ‘No tumor + HNHA’ indicates HNHA-treated mice with no xenograft; no proof of treatment-related death or systemic toxicity was observed in HNHA-treated groups (**b** and **d**). The compounds had no significant effect on mouse body weight, as compared to the control group (**e** and **f**). Photomicrographs of the dissected tumors from the treated and control mice (**g**). Weights of the dissected tumors (**h**). Immunoblot analysis of total proteins isolated from the tumors (**i**). * *P* < 0.05 vs. Control, ** *P* < 0.01 vs. Control, *** *P* < 0.005 vs. Control

All of the HDAC inhibitors tested showed significant suppression of SNU-80 and SNU-790 cell xenograft tumors; however, HNHA exhibited greater suppression of these tumors than SAHA or TSA (Fig. 5a and c). Mouse survival was extended noticeably by all of the tested HDAC inhibitors, but HNHA produced a greater effect than SAHA or TSA (Fig. 5b and d). Systemic toxicity and treatment-related deaths were not observed in any of the study groups. The body weight of mice treated with SAHA, TSA or HNHA did not differ significantly from that of the control group (Fig. 5e and f). The HNHA treatment group showed significantly smaller tumor volumes than the SAHA- or TSA-treated groups (Fig. 5g and h). The HDAC inhibitors also increased the levels of p21 (cell cycle arrest protein), GRP78 (ER stress protein) and cleaved caspase, indicating increased cell cycle arrest and apoptosis due to ER stress in these ATC and PTC mouse xenografts (Fig. 5i). These data demonstrated that HNHA produced a powerful suppression of subcutaneous thyroid cancer xenografts in an animal model.

### HNHA inhibits the proliferation of ATC and PTC xenografts in vivo

**Fig. 6:**
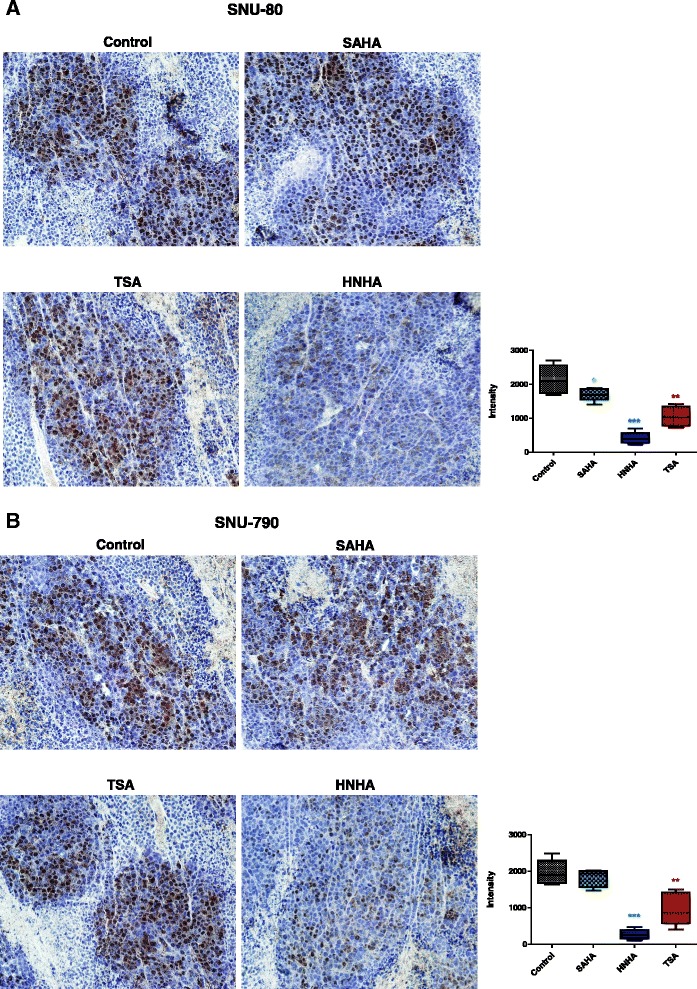
HNHA reduced tumor Ki-67 expression. Immunohistochemical analysis of the Ki-67 protein levels in paraffin-embedded tumor tissues from mice with anaplastic thyroid cancer (ATC; SNU-80; **a**) and papillary thyroid carcinoma (PTC; SNU-790; **b**) xenografts. HNHA caused more powerful inhibition of tumor Ki-67 expression than did SAHA or TSA. MetaMorph 4.6 image-analysis software was used to quantify Ki-67 immunostaining. *P < 0.05; **P < 0.01; ***P < 0.005 for the comparison with the control

Cellular proliferative activity is an important factor in the assessment of the biological behavior of carcinomas. At present, Ki-67 is the most useful marker of cell proliferation because it is expressed in all cells, except for those in the G_0_ phase. We detected this marker by immunohistochemical examination of SNU-80 and SNU-790 cell xenograft tumors and found that the HNHA-treated group showed the strongest decrease in Ki-67 expression (Fig. 6a and b). These data provided further evidence that HNHA had potent anti-thyroid cancer effects.

## Discussion

The present study showed that HNHA had potent cytotoxic effects on PTC and ATC cell lines, both in vitro and in vivo. HNHA produced a more powerful induction of apoptosis than did the other HDAC inhibitors tested in these thyroid cancer cell lines. These other HDAC inhibitors have previously been used against thyroid cancer cells [7, 24], and yet HNHA was effective at lower doses. The mechanisms underlying these cytotoxic effects of HNHA on ATC and PTC cell lines included both induction of cell cycle arrest and apoptosis. Apoptosis was demonstrated by the increased proportion of cells in sub G_1_ and by the activation of caspase 3 [25]. HNHA showed a characteristic effect on cell cycle progression, whereby G_1_ arrest was already evident in the presence of lower concentrations of HNHA, as compared to the levels of SAHA and TSA that produced this effect. This finding was consistent with those of previous studies showing that HDAC inhibitors usually produce cytotoxicity and induce G_1_ arrest at lower concentrations [21, 22]. The major molecular effect of HDAC inhibition is to change the acetylation status of core histone proteins, consequently facilitating chromatin remodeling and thus altering gene expression and cell differentiation [26–28]. Consistent with this, we found that HNHA upregulated p21 expression and downregulated cyclin D1 in the ATC and PTC cell lines. Nevertheless, although histones are regarded as the canonical acetylation substrate, some research has challenged this minimalist paradigm and indicated that HDAC inhibitors also modulated acetylation of other proteins required in an extensive range of cellular processes including protein transport, apoptosis and cell motility [29]. Histone modifications play an important role in epigenetic regulation [30] and dysregulated histone deacetylases are indicators of poor prognosis in numerous cancers. A recent research study showed that HDAC-1, -2 and -3 were highly expressed in renal cell carcinoma [31] and overexpression of HDAC1 was reported to associate with a poor prognosis [32–34]. HDAC inhibitors, which can be grouped into four structural classes, bind to the catalytic site of the enzyme and can reverse epigenetic silencing by increasing histone acetylation [35, 36].

ATC is the most aggressive type of thyroid cancer and is typically lethal, with a 1-year survival rate of just 20 % [4]. New therapies are needed to improve the prognosis of patients with this diagnosis. In this study, we have proved that HDAC inhibitors have the potential to be used for the treatment of ATC. A previous study also indicated that a different HDAC inhibitor, LBH589, modified cell cycle-controling proteins, especially cyclin D1 and p21, and powerfully inhibited the progress of ATC in a xenograft model; this was involved by a powerful decrease in Ki-67 expression in tissues from LBH589-treated animals [37].

HNHA is a dominant HDAC inhibitor that has shown strong anti-tumor activity in breast cancer in vitro and in vivo [23, 38]. Here, we demonstrated that HNHA produced more powerful anti-tumor effects than SAHA and TSA in PTC and ATC cells in vitro and in vivo, by causing apoptosis via inhibition of Bcl-2 and modulation of the cell cycle G_1_/S checkpoint signaling pathway. HNHA induced caspase-dependent apoptosis by inducing Ca^2+^ release from the ER in ATC and PTC cells, thus increasing the levels of cytoplasmic free Ca^2+^. In our study, GRP78 was noticeably upregulated in ATC and PTC cells exposed to all of the tested HDAC inhibitors. HNHA treatment also resulted in the greatest elevation of cytoplasmic free Ca^2+^ levels.

Thyroid carcinomas are generally poorly responsive to cytotoxic chemotherapy [39–42], which could be attributed to the presence of intracellular inhibitors of apoptotic signaling cascades. The present study showed that HDAC inhibitors induced pro-apoptotic proteins and reduced anti-apoptotic proteins, producing potent antitumor effects in the two thyroid cancer cell lines studied. These findings suggest that novel therapies employing HNHA alone, or in integration with usual chemotherapeutic agents, could improve outcomes in aggressive thyroid cancer. The contribution of HDAC inhibition as an anti-cancer therapy in ATC and PTC should be estimated using agents such as HNHA, which are more potent than those tested previously.

In conclusion, the anti-cancer activity of HNHA opens up a novel therapeutic approach to ATC and PTC, which do not respond successfully to conventional therapy. Translational and clinical research efforts will ultimately determine the clinical benefits and safety of HNHA, used alone or in integration with other chemotherapeutic agents, in the treatment of these types of tumor. Our findings led us to propose novel therapeutic approaches for the treatment of ATC.

## Conclusion

The current study suggests that HNHA may offer a new clinical approach to thyroid cancers, including ATC.

## Abbreviations

ATC: anaplastic thyroid cancer
Bcl-2: B-cell lymphoma-2
ER: endoplasmic reticulum (ER)
HNHA: N-hydroxy-7-(2-naphthylthio) hepatonomide
PTC: papillary thyroid carcinoma
SAHA: suberoylanilide hydroxamic acid)
TSA: trichostatin A
